# Comprehensive Transcriptome Analysis Unravels the Existence of Crucial Genes Regulating Primary Metabolism during Adventitious Root Formation in *Petunia hybrida*

**DOI:** 10.1371/journal.pone.0100997

**Published:** 2014-06-30

**Authors:** Amirhossein Ahkami, Uwe Scholz, Burkhard Steuernagel, Marc Strickert, Klaus-Thomas Haensch, Uwe Druege, Didier Reinhardt, Eva Nouri, Nicolaus von Wirén, Philipp Franken, Mohammad-Reza Hajirezaei

**Affiliations:** 1 Institute of Biological Chemistry, Washington State University, Pullman, Washington, United States of America; 2 The Sainsbury Laboratory, Norwich Research Park, Norwich, United Kingdom; 3 SYNMIKRO, Philipps-Universität, Marburg, Germany; 4 Leibniz Institute of Vegetable and Ornamental Crops (IGZ), Grossbeeren & Erfurt, Germany; 5 Department of Biology, University of Fribourg, Fribourg, Switzerland; 6 Leibniz Institute of Plant Genetics and Crop Plant Research, Gatersleben, Germany; Universidad Miguel Hernández de Elche, Spain

## Abstract

To identify specific genes determining the initiation and formation of adventitious roots (AR), a microarray-based transcriptome analysis in the stem base of the cuttings of *Petunia hybrida* (line W115) was conducted. A microarray carrying 24,816 unique, non-redundant annotated sequences was hybridized to probes derived from different stages of AR formation. After exclusion of wound-responsive and root-regulated genes, 1,354 of them were identified which were significantly and specifically induced during various phases of AR formation. Based on a recent physiological model distinguishing three metabolic phases in AR formation, the present paper focuses on the response of genes related to particular metabolic pathways. Key genes involved in primary carbohydrate metabolism such as those mediating apoplastic sucrose unloading were induced at the early sink establishment phase of AR formation. Transcriptome changes also pointed to a possible role of trehalose metabolism and SnRK1 (sucrose non-fermenting 1- related protein kinase) in sugar sensing during this early step of AR formation. Symplastic sucrose unloading and nucleotide biosynthesis were the major processes induced during the later recovery and maintenance phases. Moreover, transcripts involved in peroxisomal beta-oxidation were up-regulated during different phases of AR formation. In addition to metabolic pathways, the analysis revealed the activation of cell division at the two later phases and in particular the induction of G1-specific genes in the maintenance phase. Furthermore, results point towards a specific demand for certain mineral nutrients starting in the recovery phase.

## Introduction

Adventitious root formation (AR formation) is the key developmental process for asexual propagation of most ornamental plants. Adventitious roots arise from tissues other than the root pericycle [1] and can either be formed naturally on intact plants in dependence on the developmental program and environmental stimuli or develop in response to injury for example at the wounding site of cuttings [2]. Mostly cambium or adjacent vascular tissues are involved in AR formation in cuttings. However, our knowledge about the mechanisms that enable a somatic differentiated cell to re-differentiate into a meristematic cell to develop into a root and that laid the metabolic basis for this process is fragmentary [3], [4]. Biochemical and especially histochemical analyses revealed that AR formation should be considered as a complex multi-step process which is affected by endogenous factors, including phytohormones with a central role of auxin [5], and environmental factors, such as wounding or light [6]. In addition, the influence of carbohydrates and hormonal crosstalk [7], [8], of nitrogen supply or free amino acids [9], [10], of general mineral nutrition [11] or of antioxidative enzymes [12] on root formation has been investigated in many plants.

Focusing on physiological bottlenecks in the rooting zone of the stem base in relation to anatomical events, we defined three metabolic phases of AR formation: (1) the sink establishment phase (SEP), described by apoplastic unloading of sucrose as reflected by induced expression and high activity of cell wall invertase, (2) the recovery phase (RP), indicated by replenishment of resources and (3) the maintenance phase (MP), in which a steady state is maintained via symplastic unloading of sucrose [13]. Beside this resource-centered view based on the analysis of a limited number of genes, corresponding enzymes and metabolites, many more processes underlay such a developmental switch as AR formation and this requires changes in the expression patterns of many genes [14]. Thus, monitoring gene expression patterns is essential to understand AR formation [4].

With respect to the wide range of sugars, enzymes and intermediates shown to influence AR formation or to correlate with root formation in stem cuttings, omics-approaches aiming at describing global changes are of particular advantage. Gas chromatography-mass spectroscopy (GC-MS) and liquid chromatography-mass spectroscopy (LC-MS) have been successfully used to detect changes in metabolite contents during AR formation [15], [16], [13]. In order to achieve more knowledge on the molecular background of the biochemical and physiological processes during AR formation, it is, however, inevitable to move to the gene level and to achieve a global view on gene expression. Although adventitious rooting is a complex quantitative genetic trait regulated by both environmental and endogenous factors, the molecular mechanisms by which it is regulated are still poorly understood [6] and only a limited number of molecular studies of AR formation have been performed. For example, 220 differentially expressed genes during different developmental stages of AR formation have been identified in *Pinus contorta* hypocotyls which were treated with the auxin indole-3-butyric acid. The respective gene products were involved in protein synthesis and degradation, auxin transport, photosynthesis, cell division or cell wall synthesis [14]. Furthermore, a proteomic analysis of different mutant genotypes of *Arabidopsi*s led to the identification of eleven proteins including auxin-related and light-related proteins which positively or negatively correlated with adventitious root formation and could be suitable as molecular markers [17]. Focusing on gene expression patterns during AR formation, a number of mRNAs which were up- or down-regulated or uniquely expressed during auxin-induced adventitious root formation in apple were identified using a combination of different approaches such as differential messenger RNA display (DDRT) and mRNA representational difference analysis (RDA) [18]. Overall and based on scattered information inferred from few published studies on global gene expression and proteome analyses, the following molecular events during AR formation can be outlined in sequential order: wounding, jasmonic acid and cell wall invertase accumulation, establishment of a sink tissue, auxin transport, cell wall degradation and assembly, cell division and replication, transcription factors involved in growth and differentiation [1], [14], [6], [13], [19]. Although intensive studies on effects of single genes or metabolites during AR formation have been conducted, no conclusive models concerning the accumulation of distinct transcripts involved in various metabolic pathways during developmental stages of AR formation in leafy cuttings have been reported. Moreover, most studies have compared certain conditions (e.g. using auxin inducers) often using hypocotyl systems, rather than analyzing the temporal dynamics during spontaneous excision-induced AR formation in leafy cuttings from fully developed shoots.

The features of Petunia genus as an ornamental plant of high economic importance and its extensive uses as a model system [20] let us to establish *P. hybrida* as a model species to study molecular and physiological events in adventitious rooting of leafy stem cuttings [13], [19]. Therefore, in order to identify genes specifically induced during various developmental stages of spontaneous AR formation in leafy cuttings of *Petunia hybrida* and to describe the series of physiological processes during adventitious rooting, a microarray analysis was conducted. The microarray was described by Breuillin et al. [21] and included a normalized cDNA library from different time points after taking the cuttings. Results of the automated annotation already published were further fine-tuned and genes grouped in functional categories which could be important for AR formation. Because physiological processes and molecular changes specifically involved in AR formation were considered as of major interest, rather than those associated with wound responses, a filtration approach was chosen to eliminate primarily wound-responsive genes. The present study is mainly focused on genes related to specific metabolic pathways and will serve as a fundamental prerequisite for further functional studies.

## Materials and Methods

### Plant material, growth and harvesting conditions

Leafy stem cuttings of *Petunia hybrida* cv. Mitchell were used for all experiments. The process for production of cuttings and growth conditions were performed as described [13]. At specific developmental stages of AR formation, five mm of each cutting base (rooting zone) were harvested, immediately frozen in liquid N_2_ and stored at -80°C for further analyses. In order to determine early regulatory changes, materials were harvested at the eight most striking physiological time points (according to Ahkami et al. [13]) before any roots emerge. To simplify the description of the developmental stages, all the investigated time points are designated as hours post excision (hpe) of cuttings from donor plant: 0, 2, 6, 24, 72, 96, 144 and 192 hpe. To provide control tissues, the uppermost fully developed leaves of axillary shoots still attached at the stock plant were transversely pinched and used as wounded leaves. Samples were harvested two hours post wounding. In addition, petunia adventitious root system harvested 24 days post excision of cuttings (begin of lateral root formation) was used as fully developed root system. Three to four independent biological replicates were included for each time point and control tissue.

### Anatomical analysis

The histological examination of the material was carried out as described in detail by Haensch [22]. Briefly, cutting base segments were embedded in hydroxyethylmethacrylate (Histo-Technique-Set Technovit 7100; Kulzer, Wehrheim, Germany), cut into 6 µm slices using a Jung CM 1800 microtome (Leica Instruments, Nussloch, Germany) and stained with 0.05% toluidine blue O (Serva, Heidelberg, Germany). Microscopic analysis was performed using an AxioImager A1 microscope equipped with an AxioCam MRc5 camera (Carl Zeiss, Jena, Germany).

### Array establishment and sequence annotation

RNA was extracted from stem base (5 mm) of the cuttings at various developmental stages as described by Logemann et al. [23] and used for cDNA library construction as described [21]. Inserts were sequenced and clustered with all public available *P. hybrida* and *P. axillaris* sequences. This database of 24,816 unigene sequences was used for construction of a custom microarray [21]. All sequences of the microarray were first automatically annotated by BLASTX using the databases NCBI NRPEP and the Arabidopsis database TAIR8PEP and subsequently further manually curated and categorized using the databases PFAM for particular protein domains and BRENDA for enzymatic activities.

### Microarray hybridization and statistical analysis

Total RNA from different developmental stages of cutting stem bases and control tissues (leaves and roots) were extracted using QIAGEN kit (Qiagen, Hilden, Germany). After DNase treatment following the protocol of the DNase supplier (Qiagen), 20 µg of total RNA was used for probe synthesis and hybridization of the microarray (Roche Nimblegene, Waldkraiburg, Germany). Normalized expression values of different time points (0 hpe - 192 hpe) were compared with 0 hpe. Significantly up- or down-regulated genes were recognized via Rank Product (RP) analysis [24] using MeV (MultiExperiment Viewer, version 4.4.1.) software [25]. For each individual time point, the expression values of each replicate were divided by expression values of all four replicates at 0 hpe, followed by a log2-transformation for the Rank Product analysis (to generate M-values). Then, median values of these paired log-fold changes were calculated for each gene-related sequence identifier. Finally, the median values were back-transformed by a power of two in order to obtain the real expression ratios. The same procedure was carried out for the comparison of control tissues. To extract up or down-regulated genes, median ratios with values above two (>2) were determined as up-regulated and median ratios with values below 0.5 (<0.5) were determined as down-regulated. Putative genes showing expression value ratios more than 2 or less than 0.5 were considered as induced or repressed genes respectively (M-value >1 or <1). Based on the RP statistical analysis for each gene, using 1000 permutations, P-values below (≤0.01) were considered as significant differentially expressed. MeV software was also used to generate a plot of -Log10 of the computed P-values of the replicate effect versus the -Log10 of the P-values of the time effect based on the minimum P-values derived from Rank Product (RP) analysis according to Himanen et al. [26]. Cluster analyses (K-means clustering method) and preparation of expression graphs were performed using Genesis software version 1.7.6. [27].

### Real-Time PCR

The transcript levels of seven genes (GO_drpoolB-CL9530Contig1, cn1111, cn8317, IP_PHBS008L07u, IP_PHBS007P04u, cn3641 and cn5371) that were significantly induced during different developmental phases in the microarray experiments were confirmed by real-time PCR. RNA from three replicates of five different time points (0, 2, 6, 72 and 192 hpe) was isolated from stem bases of cuttings according to Logemann et al. [22]. The first-strand cDNA synthesis and Real-Time PCR was performed as described [18]. The mRNA levels were determined by relative quantification using actin mRNA (cn1159) as a reference related to the 0 hpe control and “2∧ -ΔΔCt” formula. Primers used are listed in Table S1.

## Results

### EST databases of genes involved in AR formation

**Figure 1:**
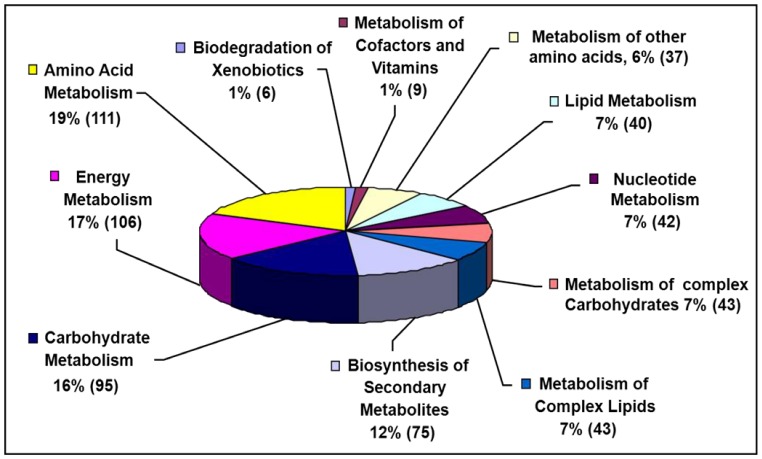
cDNA library of petunia cuttings. Classification of putative proteins into different categories of metabolism during various developmental stages of adventitious root formation in petunia cuttings. Numbers of putative proteins in different categories of the KEGG superpathway are shown in parentheses. Total protein number was 607 (out of 4,700 EST‘s).

In order to enrich the existing petunia databases by sequences from genes expressed during adventitious root formation a normalized cDNA library was generated based on pooled RNA extracted from cutting ends at particular time points (0, 2, 6, 24, 72, 96, 144 and 192 hpe). From 4700 EST's, 1,964 (42%) were singletons and 2,736 (58%) had a consensus sequence with an average sequence length of 561 bp. Annotation of sequences showed that 607 belonged to genes putatively encoding proteins which were clustered into different categories using the KEGG (Kyoto Encyclopedia of Genes and Genomes) pathway databases (Fig. 1). The largest categories were amino acid metabolism (19%), energy metabolism (17%), carbohydrate metabolism (16%) and biosynthesis of secondary metabolites (12%). More information about the generated cDNA library is available at the IPK Crop EST Databases (http://pgrc.ipk-gatersleben.de/cr-est/index.php; [28]).

### Annotation of the petunia sequence databases

Basis for the current study on AR formation in petunia was a database of 24,816 unigene sequences including the 4,700 ESTs from cuttings and a corresponding microarray which was previously used to analyse phosphate-regulation in mycorrhiza [21] (http://pgrc.ipk-gatersleben.de/petunia_array). Besides, all sequences are provided under the following URL for download: http://pgrc.ipk-gatersleben.de/petunia_array/download/. Automatic BLAST runs showed 17,282 sequences being similar to entries in the NCBI NRPEP and/or the *Arabidopsis* TAIR8PEP databases. In order to enable convenient processing of the information derived from the screening of the microarrays, one specific gene function was assigned to each unigene by curating the BLAST results. These functions were subsequently grouped into thirteen major categories (based on Journet et al. [29]) with different subgroups (Table S2a). Beside unigenes without any similarity (7,534) or those with similarity to sequences without known function (2,513) categories with most genes were ‘Protein synthesis, processing and degradation’ (2,225) signaling (1,724) and gene expression (1,683). Much lower numbers could be assigned e.g. to the categories ‘Storage’ (114) and ‘Cell Cycle’ (174) (Table S2b). The data discussed in this publication have been deposited in NCBI's Gene Expression Omnibus (according to Edgar et al. [30]) and are accessible through GEO Series accession number GSE57752 (http://www.ncbi.nlm.nih.gov/geo/query/acc.cgi?acc=GSE57752).

### Identification of genes regulated during AR formation

**Figure 2:**
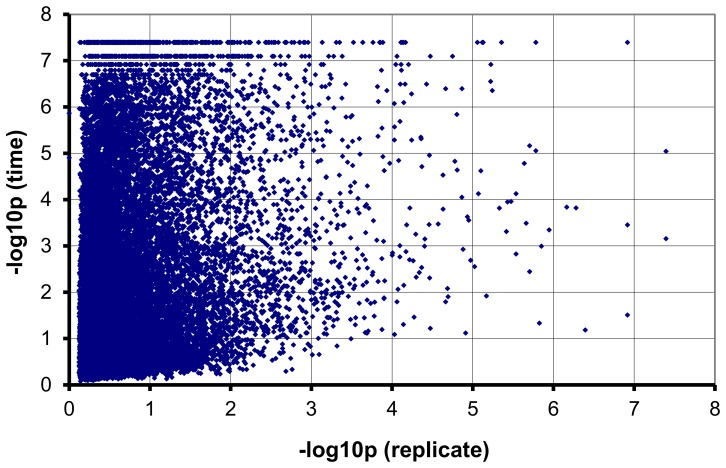
Negative correlation between the significance of time and replicate effects. Plot of -Log10 of the significance of the replicate effect (Y-axis) versus the -Log10 of the significance of the time effect (X-axis), indicating a much stronger time effect than undesired variation between replicates. Minimum P-value within replicates of all time points is located on the abscissa (X-axis) and minimum P-value of all ratios compared to 0hpe is located on the ordinate (Y-axis).

It was shown before that roots start to emerge from the stem base eight days after excision from the stock plant [13]. The rooting zone (stem base) of leafy cuttings from following eight time points during AR formation (before emergence of roots) was selected for our microarray experiment: 0, 2, 6, 24, 72, 96, 144 and 192 hpe (hours post excision of cuttings from donor plant). Histology of samples was controlled by microscopy and was similar as has been described earlier (Fig. S1), [13]. RNA was extracted from three replicates of each time point and used for array hybridization. Means of all expression values are shown in Table S3a. Determining the minimum P-values derived from Rank Product (RP) analysis revealed that only a small fraction of the genes varied in expression across the biological replicates; consequently, the reproducibility of the experiment was high. The significances of time and replicate effects, expressed as the negative 10-base logarithm of the *P* values assigned to each effect based on the Rank Product (RP) analysis, were plotted against each other (Fig. 2). The accumulation of the dots (each dot represents one sequence) on the left side of the plot illustrates the negative correlation between the significance of both effects (i.e. time and replicate), showing the high reproducibility of the expression data in this experiment. Further evidence for reproducibility came from quantitative RT-PCR. Seven genes were selected for this purpose and results coincided with the expression data observed in the microarray experiment, although, the extent of regulation was in some cases larger than deduced from microarray analysis (Table 1).

**Table 1:** Comparison of fold change differences of seven genes specifically induced during different phases of AR formation based on microarray and Real-Time qPCR.

	Fold change Microarray			Fold change qRT-PCR		
Putative function	6 h	72 h	192 h	6 h	72 h	192 h
Pectinase	−3.30	4.20	4.77	1.12±0.53	8.38±2.1	36±7.92
Glucose transporter	6.74	5.14	4.87	84.1±11.9	92.7±22	29.4±2.8
Nitrate transporter	3.21	6.91	7.12	5.0±2.0	1206±265	2645±250
F-box	6.04	2.77	2.21	32.8±0.7	15±4.3	13.8±1.5
Ubiquitin-protein ligase	1.37	3.31	3.49	3.22±0.72	7.79±3.03	6.70±2.5
Trehalose-P-phosphatase	1.93	−0.44	−0.21	1.76±0.33	1.49±0.10	0.71±0.04
Zinc/iron transporter	−0.20	7.23	7.22	0.2±0.01	166.0±47	3426±273

Each value in qPCR data is represented by the mean of three independent replicates ± standard error. Sequence ID for each gene is: pectinase (GO_drpoolB-CL9530Contig1), glucose transporter (cn1111), nitrate transporter (cn8317), F-box family protein (IP_PHBS008L07u), ubiquitin-protein ligase (IP_PHBS007P04u), trehalose-p-phosphatase (cn3641) and zinc/iron transporter (cn5371).

Based on the postulated physiological phases of AR formation [13] the following time points were selected for deriving expression ratios of all identifiers in three phases. (1) 6 hpe: representative for sink establishment (SE) phase that shows very early physiological events after excision of cuttings from donor plant when wounding leads to the establishment of a sink tissue in which all the resources are depleted through induction of genes coding for sucrose degrading enzymes in the apoplast; (2) 72 hpe: representative for recovery phase (RP) which is characterized by the formation of first new meristematic cells (dense cytoplasm and large nucleus) and the replenishment of resources; (3) 192 hpe: representative for maintenance phase (MP) as the time of appearance of first roots with vascular bundles in the center surrounded by elongated cells of the elongation zone (Fig. S1), [13]. For comparison, the rooting zone of leafy cuttings at 0 hpe, fresh leaves, wounded leaves and root tissues were used. M-values (Log2 of expression ratios) were used to demonstrate the up- and down-regulation of genes (Table S3b).

For reaching the goal of finding those genes which were specifically induced and thus may control the process of AR formation, a filtration process was carried out to remove firstly those genes linked to the inevitable wound response and secondly those genes which are related to root identity of tissues. Accordingly, genes regulated significantly during the SE phase were filtered by exclusion of those genes which were also regulated in wounded leaves versus fresh leaves. In addition, genes significantly regulated during the M phase were relieved from those genes which were also regulated in fully developed roots versus leaves. Applying such a stringent filter identified 1,354 sequences out of 24,816 identifiers (Table S3c). Since the main goal of this work was to identify genes involved in AR formation, further analyses were carried out only for these 1,354 putative genes specifically regulated during at least one of the three phases of AR formation.

**Figure 3:**
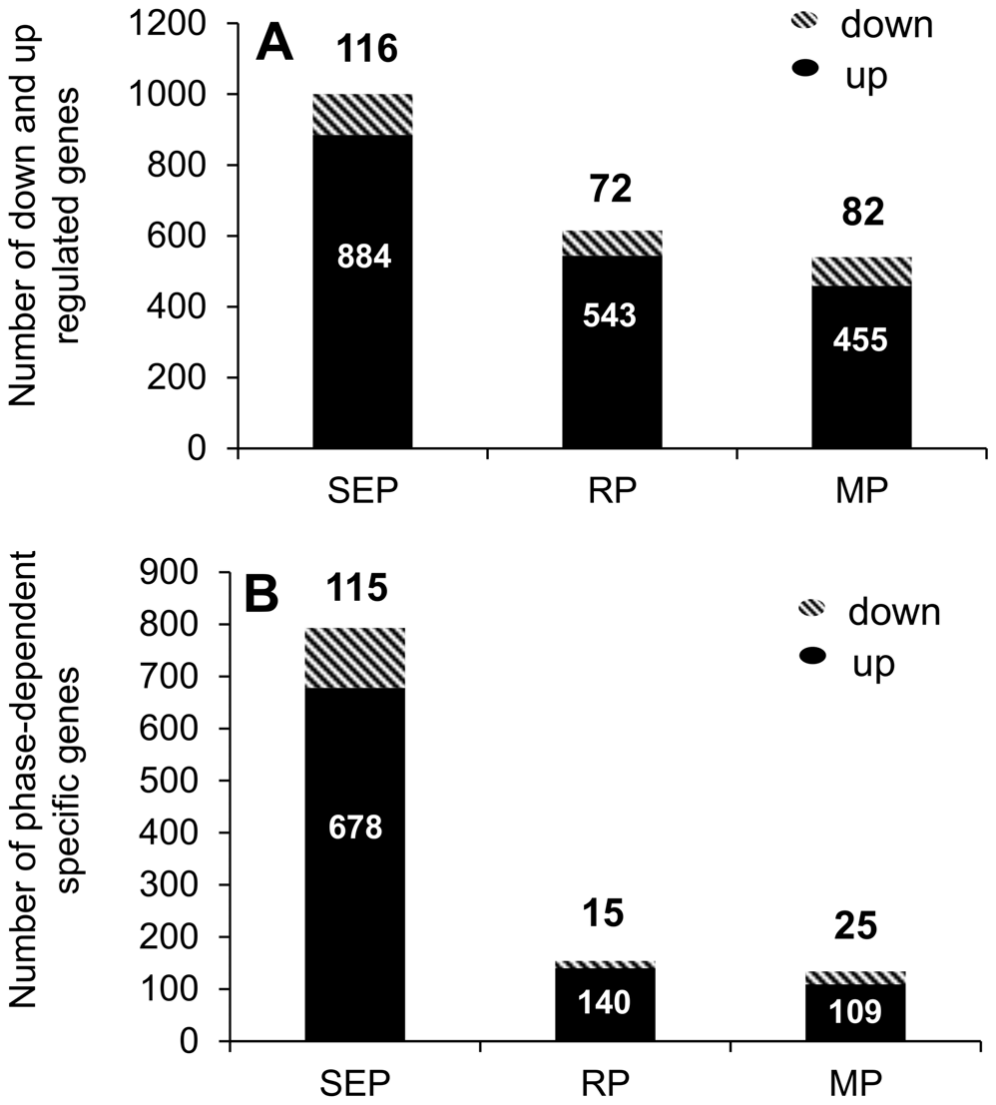
Data analysis among the 1,354 AR formation-regulated genes in petunia cuttings. (**A**) Number of up- or down-regulated genes at different dates (**B**) Number of time-dependent specific up- or down-regulated genes. SEP: Sink Establishment Phase; RP: Recovery Phase; MP: Maintenance Phase.

The analysis of differential gene expression during the three phases showed that most genes were regulated in the sink establishment phase, namely 884 were induced, and 116 repressed (Fig. 3A). Numbers of up- and down-regulated genes were observed in the maintenance and recovery phases much lower. Concerning exclusive regulation, the majority of genes (678 or 115 genes) was specifically induced or repressed during the SE phase, while the minority induced during the M phase (109 genes) and repressed during the R phase (15 genes) (Fig. 3B). Likewise, 105 putative genes were up-regulated but none down-regulated during all phases.

### Functional classification of genes specifically induced during three phases of AR formation

**Figure 4:**
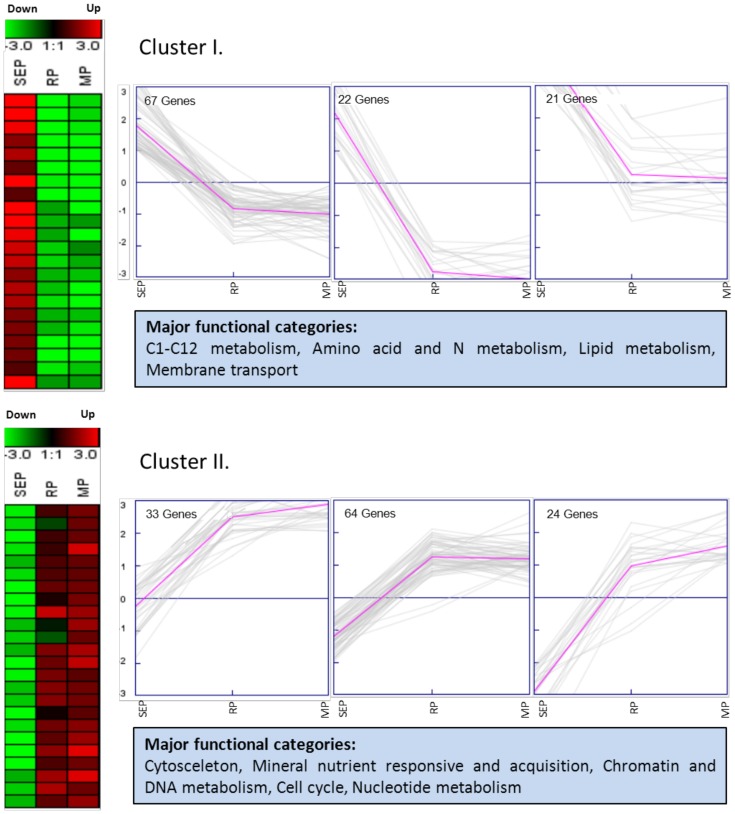
Cluster analysis. Two major clusters of genes specifically induced during different phases of AR formation created via K-means clustering method by Genesis software. Three types of associated expression graphs of each cluster are shown. SEP: Sink Establishment Phase; RP: Recovery Phase; MP: Maintenance Phase.

A total of 869 out of 1,354 genes specifically regulated during AR formation could be annotated with a known protein function (Table S3c). The majority of these genes belonged to the functional categories signaling (15.5%), protein synthesis, processing and degradation (13%), gene expression and RNA metabolism (11%), miscellaneous (8%), amino acid and N metabolism, membrane transport, C1-C12 metabolism and secondary metabolism (each approximately 5%) (Table S3c and S3d). Clustering analyses and their associated graphs showed that most genes could be classified into two major expression clusters (Fig. 4). Cluster I contained genes which were induced in the SE phase and repressed or not regulated during the other two phases. These genes putatively encode proteins involved in three subclasses of primary metabolism and in membrane transport. Genes of cluster II showed the opposite expression pattern and were induced during the R and the M phase. They encode proteins belonging to the categories cytoskeleton, mineral nutrient responsive and acquisition, chromatin and DNA metabolism, cell cycle and nucleotide metabolism.

#### (1) Sink Establishment phase (first 6 hours after excision)

Among those genes specifically induced during different phases of AR formation, 885 genes were up-regulated and 115 genes down-regulated during the first 6 hours after excision, but not by wounding. Transcripts encoding key enzymes and transporters in primary carbohydrate metabolism, such as cell wall invertase, trehalose phosphate phosphatase, trehalose-6-phosphate synthase, mannose-6-phosphate isomerase and monosaccharide transporters were induced up to 4-fold compared to 0 hpe during this time. Transcripts encoding acetyl-CoA synthetase involved in lipid metabolism were also induced early after excision. Furthermore, the transcript of proline dehydrogenase (oxidase) transcript which catalyzes the first step in proline catabolism in mitochondria was up-regulated about 3-fold in this phase, whereas transcripts encoding zinc/iron transporters and phosphate transporters were down regulated or remained unchanged during the first six hours after excision. Moreover, a transcript encoding ribonucleotide reductase was repressed, while a 3-hydroxyacyl-CoA dehyrogenase-encoding gene involved in β-oxidation was up-regulated in this phase. As components of signaling pathways, transcript abundances of sucrose non-fermenting 1- related protein kinase (SnRK1), transducin family protein/WD-40 repeat family protein and inositol-1,4,5-triphosphate-5-phosphatase were up-regulated up to two-fold only in this phase. Focusing on amino acid and N metabolism, the results showed an approximately 2-fold up-regulation in the expression of a transcript encoding S-adenosylmethionine synthase (SAMS) during SE phase.

#### (2) Recovery phase (3 days after excision)

**Figure 5:**
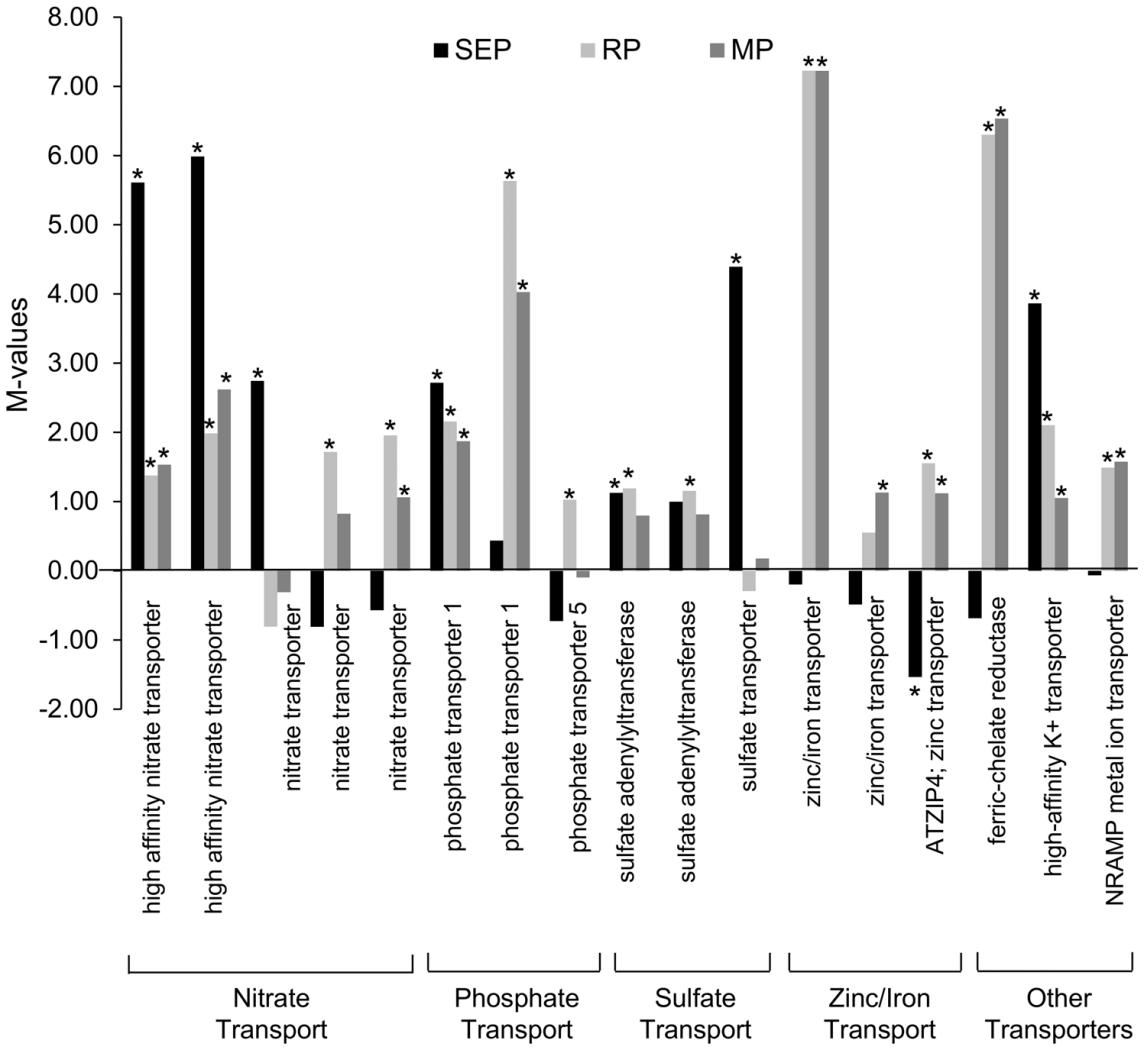
Fold change ratios of mineral nutrient transporters during AR formation in petunia cuttings. M values (Log2 of fold change ratios) of genes involved in mineral nutrient transport and processing during various phases of AR formation in petunia cuttings compared to the time point 0 hpe. SEP: Sink Establishment Phase; RP: Recovery Phase; MP: Maintenance Phase. Sequence ID for each gene in sequential order as illustrated in the figure: high affinity nitrate transporters (GO_drpoolB-CL2736 Contig1, GO_drs31P0006M14_R_ab1), nitrate transporters (cn1356, cn7095, GO_drpoolB-CL8921Contig1), phosphate transporter 1 (cn1272, cn8235), phosphate transporter 5 (cn8236), sulfate adenylyltransferases (GO_drpoolB-CL2256Contig1, GO_drpoolB-CL4792Contig1), sulfate transporter (cn6307), zinc/iron transporters (cn5371, GO_dr004P0016I22_F_ab1), ATZIP4 zinc transporter (IP_PHBS009K04u), ferric-chelate reductase (GO_drpoolB-CL1069Contig 1), high-affinity K+ transporter (cn7074), NRAMP metal ion transporter (GO_drpoolB-CL1711Contig1). Significant changes are indicated by asterisks.

Among the genes specifically induced during AR formation, 544 genes were up-regulated and 71 genes down-regulated three days post excision. Transcripts involved in primary metabolism such as phosphoenolpyruvate (PEP) carboxylase, glutamine synthetase and enoyl-CoA hydratase were up-regulated at 72 hpe. However, transcripts encoding intermediates involved in trehalose metabolism were no longer induced in this phase as well as apoplastic invertase. Furthermore, genes involved in chromatin and DNA metabolism, cytoskeleton, cell cycle (cell division related genes, CycB1, ribonucleotide reductase, uridine 5′-monophosphate synthase) and mineral nutrient transport started to be highly up-regulated in this developmental stage. In particular, phosphate transporter (PT) and zinc/iron transporter-encoding (Zn/Fe-T) genes showed about up to five and seven-fold up-regulation, respectively (Fig. 5). In addition, a transcript of peroxidases,1-pyrroline-5-carboxylate dehydrogenase (P5CDH) involved in proline metabolism was also induced in this phase. Focusing on signaling-related genes, RNA levels of Ran GTPase activating protein were increased three days after excision.

#### (3) Maintenance phase (8 days after excision)

A group of 459 genes were up-regulated and 81 genes down-regulated eight days after excision of the cuttings from the stock plant, but were not higher expressed in roots than in leaves. In general, most genes showed a similar expression pattern in the R and M phases. Likewise, transcripts encoding important mineral nutrient acquisition genes, such as transporters for metals, zinc/iron, phosphate or nitrate, were highly up-regulated up to 7-fold at 192 hpe (Fig. 5). Furthermore, different types of peroxidases, P5CDH, UMP synthase, and cyclin family of cell cycle regulators accumulated at 72 hpe and continued to remain at an elevated level (about 2-fold) until 192 hpe. Interestingly, transcripts encoding various types of Cyclin D were induced up to approximately 1.5-fold only in M phase. In addition, transcripts encoding extensin (component of plant cell walls) and a plasma membrane proton ATPase were up-regulated in this phase, exclusively.

## Discussion

A cutting removed from the donor plant undergoes various anatomical changes accompanied by alterations in gene expression patterns during the wound response and subsequent rhizogenesis. To investigate the occurrence and temporal sequence of specific gene expression changes during AR formation, different time points after taking the cutting were analysed using the model plant *Petunia hybrida*. These time points exemplified the three metabolic phases which have been identified before [13]. To identify candidate genes specifically regulated during AR formation a microarray analysis was carried out. In order to monitor non-disturbed physiological events during spontaneous excision-induced AR formation in full dependence on the initial condition of the cutting, no chemicals were applied to the substrate. Likewise, fresh leaves were compared with wounded leaves and with fully developed roots to eliminate those genes that are not specifically related to AR formation. Out of 24,816 detected sequences, 1,354 were finally considered as AR formation-regulated genes of which 869 genes putatively encoded proteins with known functions. The predicted proteins were clustered according to the process in which they are involved. With regard to the large number of genes specifically regulated during AR formation, a focus was set on key genes involved in primary metabolism, signaling, cell replication and mineral nutrient acquisition. Among the regulated genes 355 encode proteins with unknown functions. This provides a potential source to identify new candidates for adventitious rooting by means of reverse genetics.

### Distribution of identified genes among metabolic pathways during AR formation

A three-phase mechanism for the metabolic response involved in AR formation in petunia has been postulated [13]. During the first hours post excision, i.e. sink establishment phase, assimilates are produced in source tissues and translocated towards the cutting base to establish a sink organ. This event was associated with accumulations of transcripts coding for cell wall invertase leading to cleavage of sucrose by this enzyme within the apoplast and transport of the products (glucose and fructose) into the cell by monosaccharide transporters. Two days after excision, the recovery phase starts that is identified by the replenishment of resources ending with the formation of meristemoids. This stage is followed by the maintenance phase characterized by symplastic transport of sugars translocated from source leaves into the stem base used either for energy production (as intermediates required directly for root development) or accumulated in the vacuole (as intermediate storage compounds utilized for later root formation processes) [13].

Based on the data derived from gene functional classification of the current study, different genes were shown to be up- or down-regulated in three phases of AR formation in different metabolic pathways which interestingly supported our previous data and additionally revealed new findings.

### Primary metabolism

Approximately 17% of the known and functionally classified AR formation-regulated genes were related to primary metabolism. Except those involved in nucleotide metabolism, the majority was induced during the SE phase followed by a repression afterwards.

#### Sucrose unloading

Transcript levels of apoplastic (cell wall) invertase and monosaccharide transporters accumulated at 6 hpe followed by a reduction during the recovery and maintenance phases. This is a possible indication for a switch from apoplastic to symplastic phloem unloading of sucrose during AR formation and is in agreement with previous findings [13]. Carbohydrates may have to be imported into the affected tissues to avoid the risk of energy limitation, and this could be accompanied by a rapid and simultaneous induction of genes coding for extracellular invertases [31] or putatively plasmalemma-localized monosaccharide transporters [32]. As the supply of the main transport sugar, i.e. sucrose, is a limiting step for the growth and metabolism of sink tissues [33], better understanding of sucrose efflux from source tissue, its degradation and translocation within phloem towards the stem base in petunia cuttings will contribute to obtaining more comprehensive insight of the growth and development of adventitious roots which eventually will lead to successful manipulation of carbohydrate partitioning and thus to an improvement of rooting ability of petunia cuttings.

#### Glycolysis

The transcript levels of three out of total ten enzymes involved in glycolysis were specifically induced during AR formation in petunia cuttings. This observation is in agreement with biochemical results regarding the enzymes involved in glycolysis [13]. These included genes for hexokinase (HXK) and of three isoforms of phosphofructokinase (PFK) and of phosphoglycerate mutase (PGM). Induction of HXK catalyzing the conversion of hexoses to hexose-phosphates and PFK that catalyzes the conversion of fructose-6-phosphate to fructose-1,6-bisP indicates that carbon flux through glycolysis is important during AR formation. Except fundamental roles of glycolysis [34], in recent years, additional non-glycolytic functions such as regulation of transcription have also been attributed to glycolytic enzymes [35].

#### Trehalose metabolism

Trehalose metabolism, a short side-branch of primary carbon metabolism which is controlled by a large gene family, is emerging as an important new regulatory pathway in plants [36]. In the SE phase transcripts encoding two key enzymes involved in trehalose metabolism including trehalose-6-phosphate synthase (TPS) and trehalose-6-phosphate phosphatase (TPP) were induced. Trehalose-6-phosphate (T6P), essential for the coordination of metabolism with plant growth adaptation and development [37], is formed from glucose-6-phosphate and uridine-5-diphosphoglucose (UDPG) by TPS and is then dephosphorylated to trehalose by TPP [38]. Subsequently, trehalose may be converted to glucose by trehalase. TPS1 is expressed at low levels in all tissues, peaking in metabolic sinks such as embryos, flower buds, young rosette leaves [39] or in the stem base of petunia cuttings in the current study. However, Schluepmann et al. [40] presented evidence that the active component of the trehalose pathway that regulates metabolism is T6P. In the same direction, Paul et al. [41] suggested that T6P either directly or indirectly controls carbon assimilation. According to Kolbe et al. [42] T6P may directly activate ADP glucose pyrophosphorylase (AGPase), the key enzyme in starch synthesis. Sucrose leads to the redox activation of AGPase in a sucrose non-fermenting 1-Related protein Kinase (SnRK1)-dependent manner when fed to intact leaves [43]. SnRK1 is a protein kinase involved in the regulation of carbohydrate metabolism. In the current investigation, a SnRK1 homolog for petunia was induced in the sink establishment of AR formation. When micromolar T6P was fed to isolated chloroplasts, it activated AGPase, providing the possibility that T6P is the conduit for sucrose and possibly trehalose-mediated activation of starch synthesis [37]. In this way, T6P can be seen as a sugar signal that communicates the sucrose status of the cytosol to the chloroplast [37]. It has further been suggested by Lunn et al. [44] that T6P acts as a signaling metabolite of the sugar status in plants, and that it mediates sucrose-dependent changes in the rate of starch synthesis. Besides, it has been reported that T6P is associated with the expression of SnRK1 [45]. Taken together, it may be concluded that T6P, the product of TPS, along with SnRK1 may have a signaling effect on starch biosynthesis and accumulation in the later phases of AR formation. Accumulation of starch in the later phases of AR formation in petunia cuttings has been reported which probably acts as the major carbon source when the adventitious roots grow [13]. Additionally, induction of a plastidic aldolase gene in the recovery phase in the current study is also indicative of starch biosynthesis in the chloroplast.

#### Lipid metabolism

The present transcriptome study suggests an active lipid metabolism in petunia cuttings especially during the SE phase. Transcript levels of two enzymes involved in plant peroxisomal fatty acid β-oxidation including 3-hydroxyacyl-CoA dehyrogenase and enoyl-CoA hydratase were induced during early and later stages of AR formation, respectively. Beyond its role in the breakdown of storage lipids, β-oxidation in plants has been shown to be active in a variety of other developmental processes, including the emergence of the radicle from the seed coat, embryo and flower development, production of jasmonic acid (JA) in the wound response or generation of the phytohormone indole-3-acetic acid (IAA) [46]. The fact that AR formation is a developmental process which utilizes carbohydrates as a main carbon source for energy production [13] and requires auxin for its initiation step [18], suggests that degradation of reserve lipids by β-oxidation is possibly used for JA synthesis and for the conversion of natural auxin indole-3-butyric acid (IBA) into IAA during AR formation in petunia cuttings.

#### Other induced genes involved in primary metabolism

Besides its cardinal roles in the initial fixation of atmospheric CO_2_ during C_4_ photosynthesis and Crassulacean Acid Metabolism (CAM), PEP carboxylase (PEPC) functions anaplerotically in a variety of non-photosynthetic systems such as for C/N partitioning in C_3_ leaves, seed formation and germination, or ripening [47]. The results showed that PEPC transcripts were induced during the recovery phase. This is accompanied by an increased PEPC enzyme activity during the same phase [13] which may confirm that PEPC has a role in refilling TCA cycle during later phases of AR formation. Likewise, consistent with a proteome analysis of adventitious rooting in *Arabidopsis* [17], RNA levels of carbonic anhydrase (CA) were induced at 6 hpe in this study. The corresponding enzyme functions in the production of bicarbonate, which serves as a substrate for PEPC [48]. The product of PEPC activity, oxalacetate, can be converted to aspartate via aspartate aminotransferase (AAT) [48], which was also induced at the transcript level at the same time point in the current study. This observation is supported by a continuous increase of aspartate levels in the petunia cuttings three days post excision [13]. Thus, CA, PEPC and AAT may represent regulatory steps in the pathway for the synthesis of aspartate during AR formation.

The degradation of proline is catalyzed by the sequential action of two mitochondrial enzymes, Pro-dehydrogenase (ProDH) and P5C-dehydrogenase (P5CDH). Transcript levels of ProDH, also known as proline oxidase (POX), which is catalyzing the rate-limiting oxidation/dehydrogenation of proline to pyrroline-5-carboxylate (P5C) [49] was up-regulated in the sink establishment phase followed by a reduction at later time points. In contrast, lower transcript levels of P5CDH, catalyzing the conversion of P5C to glutamate, in the SE phase was followed by an acceleration in the R and M phases. These results suggest that the generated P5C in the SE phase may have been further converted to glutamate in the later phases. Consistently, the amount of glutamate has been shown to be increased at the beginning of the R phase (72 hpe) in the stem base of petunia cuttings [13]. Nevertheless, these data are not in agreement with proline accumulation in M phase [13]. The apparent discrepancy between high proline concentrations and high ProDH/P5CDH (proline degradation enzymes) transcripts levels may be explained by post-transcriptional regulation of the corresponding proteins or by proline export via the xylem to the stem base in the M phase. Considering the role of proline transport, Raymond and Smirnoff [50] implied that changes in biosynthesis and oxidation rate which led to proline accumulation are at least partly controlled by changes in gene expression and enzyme activity. A previous conflict between low proline concentrations and high P5CS/P5CR (proline synthesizing enzymes) transcripts levels in roots was explained by proline export via the xylem to the shoot [51]. Proline has been suggested to act as a compatible osmolyte and as a storage carbon and nitrogen compound [52]. In this regard, Schwacke et al. [53] proposed that the increase of compatible solutes is achieved either by altering metabolism (increasing biosynthesis and/or decreasing degradation) or by transport (increased uptake and/or decreased export). Moreover, proline transport to flowers is also active, as it can be a compatible solute to transfer nitrogen, carbon and energy to developing flowers and seeds [54]. Finally, proline accumulation may be part of a stress signal cascade influencing adaptive responses [55]. The hypothesis of proline transport to the stem base of petunia cuttings at the later stages of AR formation and its possible role as a compatible osmolyte should be further investigated.

A transcript encoding S-adenosylmethionine synthase (SAMS) was also induced in the SE phase, which is in agreement with earlier investigation showing that two SAMS-encoding genes were induced during adventitious root development in *Pinus contorta* [56]. In addition, several sequences showing homology to SAMS were also up-regulated during AR formation in *P. contorta* [14]. SAMS catalyzes the production of S-adenosylmethionine (SAM) which has a central position in several physiological processes [56]. In plants, SAM serves as a methyl group donor in the transmethylation of lignin, DNA and alkaloids, or as a donor of aminopropyl moieties in ethylene and polyamine synthesis [57], [58], [59]. Regarding the fact that the expression of this enzyme is not generally up-regulated under stress conditions [60] and considering the consistent results in previous investigations for AR formation, SAMS could be regarded as a significant candidate gene which may play an important role specifically during the rooting of cuttings.

### Cell division

In total, 29 genes specifically induced during AR formation in petunia cuttings are involved in chromatin and DNA metabolism. The number of up-regulated genes in this category was higher in the R and M phases of AR formation in comparison to the first six hours after excision, while no genes were down-regulated. The same expression pattern was observed for a total of 15 genes involved in the class of cytoskeleton-related functions. In addition, the number of up-regulated genes involved in cell cycle processes increased during AR formation, while the opposite trend was observed for down-regulated genes. These results indicate an increase in the induction of chromatin and DNA metabolism, cell cycle and cytoskeleton-related genes starting 72 hours after excision which coincided with the previous reports of formation of new meristematic cells at 72 hpe in the stem base of petunia cuttings [13], [19]. For example, two genes encoding histon H_4_ were up-regulated in the R and M phases indicating an induction of the cell cycle during the late phases of AR formation. Similar observations were reported for AR formation in *Pinus contorta* [61], [14] and rice [62]. Concerning the cell cycle, transcripts encoding cyclin B1 were induced three days after excision and thereafter. These types of cyclins are regulatory proteins of cyclin-dependent protein kinases. Northern blot analysis of the *cyclin B1* gene showed a similar time-dependent pattern during AR formation [13]. Interestingly, transcripts encoding cyclin D were only induced in the M phase. D-type cyclins regulate the progression of cells through the G_1_ phase of the cell division cycle in response to extracellular signals such as auxin, cytokinin or sucrose [63]. Thus, we suggest *cyclin D* as a specific marker for the M phase of AR formation in petunia cuttings.

Regarding ribonucleotide synthesis, a transcript encoding ribonucleotide reductase (RNR) was down-regulated in the sink establishment phase followed by an up-regulation in the recovery and maintenance phases. RNR catalyzes the rate-limiting step in the production of deoxyribonucleotides needed for DNA synthesis [64]. A critical role of RNR in cell cycle progression, DNA damage repair, and plant development has been reported [65]. In coincidence with the formation of the first new meristematic cells at 72 hpe (Fig. S1), [13], up-regulation of RNR in the recovery phase of AR formation may be expected. In the same direction, a transcript encoding uridine 5′-monophosphate synthase (UMP synthase), which is the rate-limiting step in pyrimidine biosynthesis [66], was up-regulated in the recovery and maintenance phases. Therefore, the recovery phase is characterized by the induction of genes involved in ribonucleotide synthesis, such as RNR and UMP synthase.

### Mineral nutrient acquisition

Nutrition is a key factor of root morphogenesis through effects on primary root length or lateral root formation [11]. Although adventitious rooting and mineral nutrition are intimately related, only a few studies have attempted to characterize the action of certain mineral elements on the rooting process. However, recently Santos et al. [67] reported that nutrient availability at the stem base of petunia cuttings at root emergence phase improves AR formation. In the current transcriptome analysis in petunia cuttings, a total of 18 mineral nutrient related-genes were specifically induced during root development, in particular at later phase of AR formation. The fold change ratios of mineral nutrient transporters are illustrated in Figure 5.

Transcript levels of several nitrate transporters (NT) were induced during AR formation. Some transporter genes were induced in the SE phase (up to 5-fold) and also in the R and M phases (Fig. 5). The induction of NTs at 6 hpe most likely indicated nitrogen deficiency of the cuttings early after excision. Besides being a source of nitrogen, nitrate serves as a metabolite to buffer acidification from ammonium assimilation and as a signal for growth [68], [69]. Induction of NTs at later stages of AR formation is consistent with the well-established role of some types of nitrate transporters in regions containing rapidly dividing cells [70]. Besides their metabolic role of transporting nitrate in nascent organs, the activity of NTs may contribute to nitrate signaling or pH homeostasis during lateral root formation [70].

Plants have evolved sophisticated metabolic and developmental strategies to maximize inorganic phosphate (P_i_) acquisition when P_i_ is limited. One of the main strategies is to increase the expression of high-affinity P_i_ transporters [71]. In the current study, transcripts encoding three phosphate transporters were induced at different time points of AR formation with the trend of a preferential up-regulation at later stages. Phosphate is necessary for various developmental and physiological processes such as root formation, cell division and nucleotide synthesis [72].

Transcript levels of Zn and Zn/Fe transporters were also over-represented in the R and M phases. These micronutrients may function as cofactors for enzymes required during AR formation in petunia cuttings, such as ribonucleotide reductase or fatty acid desaturase which are Fe-dependent or carbonic anhydrase which uses Zn as its prosthetic group. Moreover, Zn is required for the biosynthesis of tryptophan which is an auxin precursor [73], [74]. In agreement with this, high Zn concentrations in the induction phase of adventitious root formation in microcuttings of *Eucalyptus globulus* influenced auxin concentrations, thereby favoring the rooting response [11]. Since Fe participates in the biosynthesis of peroxidases [75], increase of Fe transporters, which are at least one of the requirements to accelerate Fe levels, during the later phases of AR formation could cause an enhancement of peroxidase activation possibly involved in auxin catabolism. A similar explanation was suggested by Fang and Kao [76] as they implied Fe reduction in rice leaves could cause a decrease in the activities of peroxidases. Besides, decreasing Fe concentrations in the induction phase of adventitious rooting in microcuttings of *Eucalyptus globulus* showed only a trend towards higher root number and longer root length [11]. However, Fe may also act on other root morphological traits via micronutrient signaling effects that also interfere with hormonal regulation [77]. Moreover, in the maintenance phase, a high transcript abundance was observed for a gene encoding a plasma membrane proton ATPase, which is most likely contributing to cell elongation [78] or nutrient uptake [79].

### Antioxidative metabolism and Redox state

Four out of 18 genes on the microarray that are involved in antioxidative metabolism encoded peroxidases indicating their possible important functions in root development. Three were up-regulated in the R and M phases, between 1.5 to 3-fold. One isoform of peroxidase has been reported as a predictive marker of AR formation in *Betula pendula* [80]. Besides, changes in peroxidase activity and peroxidase isoform patterns have been proposed as biochemical markers of successive rooting phases [12], [81]. However, they have also been implicated in several physiological processes including the regulation of growth, cell expansion [82], lignification [83] and interestingly also in auxin catabolism [84]. This could be an important function at later stages when auxin becomes inhibitory in AR formation [19], [85].

### Summary and conclusion

**Figure 6:**
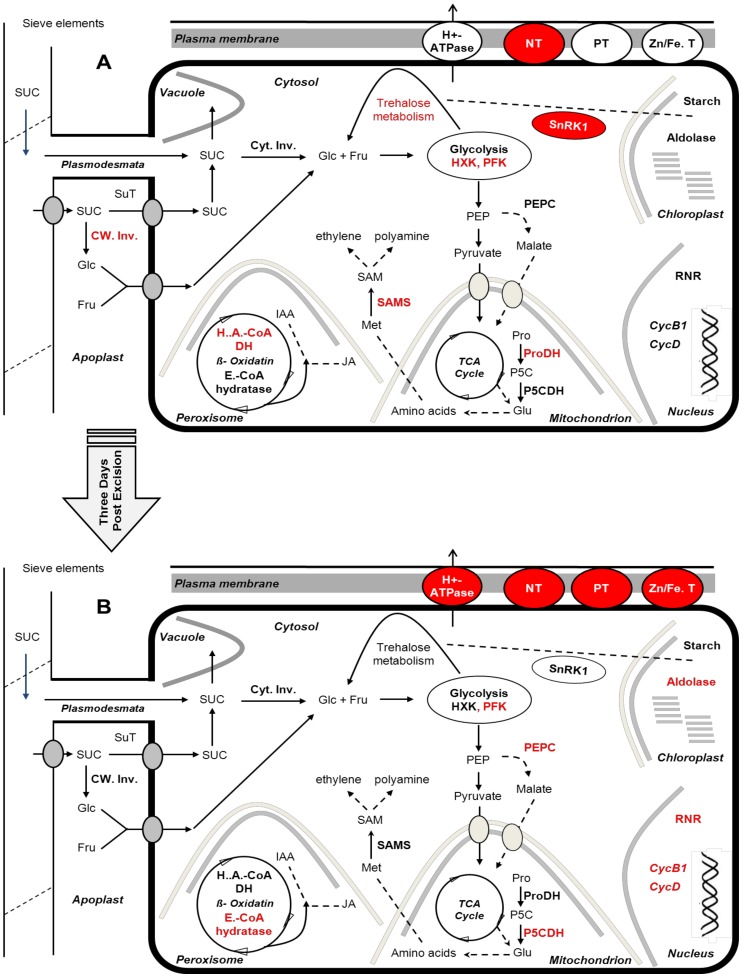
Schematic illustration of metabolic pathways regulated during AR formation in petunia cuttings. In the figure, only important induced key genes in primary metabolism, cell replication machinery, signalling and membrane transport are included. Induced genes are shown in red color. Because the observed gene expression patterns in R and M phases were not significantly different and to simplify the figure, only two phases have been illustrated. (**A**) SE phase (**B**) R and M phases. NT: nitrate transporter, PT: phosphate transporter, Zn/Fe.T: zinc/iron transporter, Cyt. Inv: cytosolic invertase; CW Inv: cell wall invertase; SuT: sucrose transporter; PFK: phosphofructokinase, HXK: hexokinase, RNR: ribonucleotide reductase; PEPC: phosphoenolpyruvate carboxylase; H.A.CoA DH: 3-hydroxyacyl-CoA dehydrogenase; E.CoA hydratase: enoyl-CoA hydratase; Pro: proline; ProDH: proline dehydrogenase; P5CDH: pyrroline-5-carboxylate dehydrogenase; Met: methionine; SAMS: S-adenosylmethionine synthase; SAM: S-adenosylmethionine.

By examining changes in the global gene expression in petunia stem cuttings, a temporal sequence of molecular events taking place during adventitious rooting was characterised. While changes in mRNA levels do not necessarily lead to alterations in protein levels, the importance of transcriptional studies as a critical regulatory level of biological systems has been well established [86], [87]. Based on the presented data and focusing on changes in transcript abundances of genes involved in primary metabolism, membrane transporters, cell division or signalling during various phases of AR formation following working model has been set up (Fig. 6).

During the sink establishment phase, carbohydrate metabolism in the stem base is initiated by an increase of cell wall invertase and of hexose transporters to import the products of sucrose degradation from the apoplast into the cytosol. Simultaneously, several genes involved in trehalose metabolism are up-regulated which may regulate carbohydrate metabolism in concert with SnRK1 in order to convey the sucrose status of the cytosol to the chloroplast. Transcripts encoding PFK and HXK are increased showing active glycolysis during the sink establishment phase. Key genes involved in nucleotide biosynthesis and in the cell cycle are repressed indicating that cell division is not a cellular process required during the sink establishment phase. In addition, a SAMS-encoding gene suggested functioning as an intermediate in ethylene or polyamine biosynthesis is active. Peroxisomal beta oxidation seems to be active at least by induction of one associated enzyme, indicating its possible role in JA or IAA production (Fig. 6A).

Three days post excision, the cell replication machinery is up-regulated via induction of CycB1, RNR and UMP synthase coinciding with the formation of new meristematic cells. Transcript levels of extracellular invertase and monosaccharide transporters are decreased indicating a switch from apoplastic to symplastic sucrose unloading. An activation of genes necessary for mineral nutrient acquisition takes place and glutamine synthetase is up-regulated in order to stimulate ammonia assimilation. PEPC is induced to refill the TCA cycle or to support aspartate biosynthesis. Peroxisomal beta oxidation is supposed to be still active as indicated by the induction of enoyl-CoA hydratase. The transcript level of a plastidic isoform of fructose-bisphosphate aldolase is up-regulated suggesting active glycolysis in the chloroplast which may support starch biosynthesis. Further, the generated P5C in the sink establishment phase is now converted to glutamate through P5CDH activation in mitochondria (Fig. 6B).

In general, most of the observed expression patterns in the R phase were continued in the M phase (Fig. 6B), suggesting 72 hpe as a central time point at which developmental processes switch or initiate. However, the development of non-emerged but fully differentiated roots, eight days post excision, is accompanied by an activation of G_1_ phase-specific genes of the cell cycle as indicated by the up-regulation of Cyclin D. The plasma membrane H^+^-ATPase may be up-regulated to support cell elongation or nutrient uptake. The induction of transcripts encoding genes involved in cell division, and mineral nutrient acquisition is continued. In comparison to the recovery phase, an enhancement of transcript levels for extensin was monitored supporting the idea that the cell replication machinery is more active shortly before root emergence.

Taken together, several candidate genes have been identified through the global transcriptome analysis that might play a critical role in AR formation and require further investigations for functional analysis by generating transgenic lines through sense or anti-sense approaches. These include genes involved in trehalose metabolism (TPP and TPS), beta oxidation pathway, mineral nutrient acquisition, sucrose unloading process as well as SnRK1 or SAMS.

## Supporting Information

Figure S1 **Anatomy of adventitious root formation in the stem base of the examined Petunia cuttings.** (**A, B**) Different magnifications of a cross section at 72 hpe showing the cortex (co), the pith parenchyma (pi) and a ring of vessels (r) with the outer phloem (oph), the cambium (ca), the xylem (xy), the inner phloem (iph) and the first meristematic cells (mc) of developing root meristems, i.e. small cells with a dense cytoplasm and large nuclei. (C–F) Cross sections, revealing at 96 hpe (**C**) first developing root meristems (me), at 144 hpe (**D**) first differentiating root primordia with an organized meristem and a backward differentiation of cells of the root body (root cortex (ro) and vascular bundle (v)) and at 192 hpe (**E, F**) first roots with vascular bundles (v) surrounded by elongated cells (ec) of the elongation zone and root hairs (rh). Indicated bars represent 500 µm for A and E, 200 µm for F and 100 µm for B, C and D.
(TIF)

Table S1 **Gene-specific primers for Real-Time qPCR.** Analysis was carried out using actin as housekeeping gene. All Real-Time qPCR reactions were repeated three times.
(DOCX)

Table S2 **Annotations and categories.** (**S2a**) Annotations: Based on BlastX searches in the NCBI and the TAIR peptide non redundant databases (published in Breuillin et al. 2010) and on information of the Pfam protein family and the BRENDA enzyme database, one particular function was annotated to each sequence identifier and classified into functional categories. (**S2b**) Category abundance: Number of genes grouped in different functional categories are shown.
(XLSX)

Table S3 **Expression values and ratios.** (**S3a**) All expression and corresponding mean values. Arrays were hybridized to complex cDNA probes derived from stem bases 0 hours up to 192 hours post excision (hpe) of the cutting as well as from fresh leaves (FrL), from wounded leaves (WL) and from a root system (Root). Shown are means of expression values from 3 to 4 biological repetitions. (**S3b**) Expression ratios for all sequence identifiers. M-values (Log2 of expression ratios) are shown for comparisons of expression values of the Sink establishemnt phase (6 hpe), the Recovery Phase (72 hpe) and the Maintenance Phase (192 hpe) to the expression values at 0 hpe, as well as the comparison of fresh leaves (FrL) to wounded leaves (WL) and from root systems (Root) to cuttings at 192 hpe. M-values for putative genes being more than 2-times induced or repressed and where the differences are significant according to Rank Product Analysis are highlighted in red or in green, respectively (M-value >1 or <1). (**S3c**) Expression ratios for all AR formation-specifically regulated putative genes. M-values (Log2 of expression ratios) are shown for thos sequence identifiers belonging to putative genes induced or repressed during the Sink establishment phase (6 hpe) and not regulated by wounding, and during the Recovery Phase (72 hpe) or the Maintenance Phase (192 hpe) and not differentially expressed in the comparison roots versus shoot basis. M-values for putative genes being more than 2-times induced or repressed and where the differences are significant according to Rank Product Analysis are highlighted in red or in green, respectively (M-value >1 or <1). (**S3d**) Number of down- or up-regulated genes specifically induced during different phases of AR formation in petunia cuttings in each individual category. SEP: Sink Establishment Phase, RP: Recovery Phase, MP: Maintenance Phase.
(XLSX)
