# Supplementary figures and images for: Comprehensive Transcriptome Analysis Unravels the Existence of Crucial Genes Regulating Primary Metabolism during Adventitious Root Formation in Petunia hybrida
Source: PLoS One. 2014 Jun 30;9(6):e100997. doi: 10.1371/journal.pone.0100997 (PMC4076263; doi:10.1371/journal.pone.0100997)

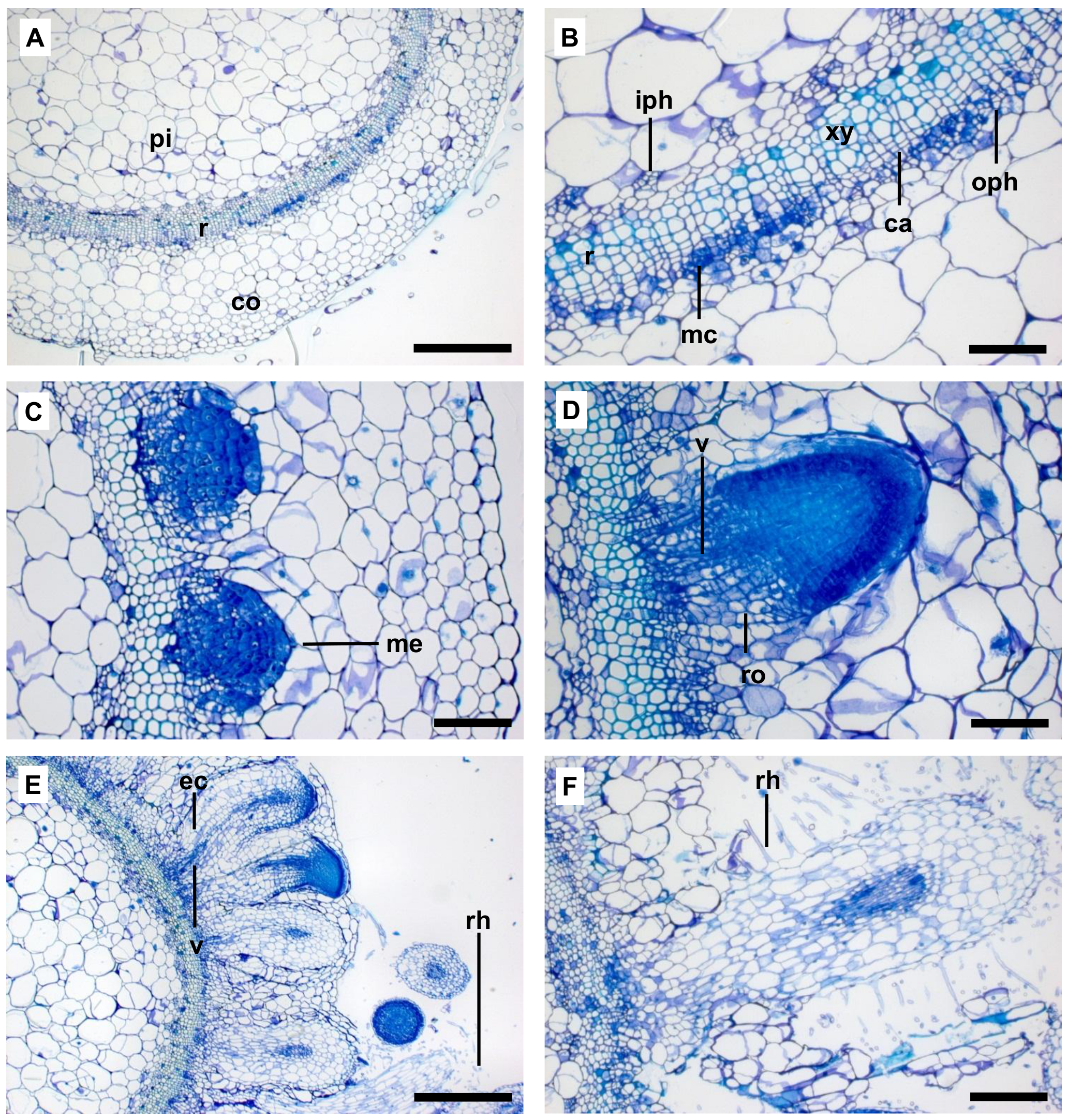

Supplement: Figure S1 — Anatomy of adventitious root formation in the stem base of the examined Petunia cuttings. (A, B) Different magnifications of a cross section at 72 hpe showing the cortex (co), the pith parenchyma (pi) and a ring of vessels (r) with the outer phloem (oph), the cambium (ca), the xylem (xy), the inner phloem (iph) and the first meristematic cells (mc) of developing root meristems, i.e. small cells with a dense cytoplasm and large nuclei. (C–F) Cross sections, revealing at 96 hpe (C) first developing root meristems (me), at 144 hpe (D) first differentiating root primordia with an organized meristem and a backward differentiation of cells of the root body (root cortex (ro) and vascular bundle (v)) and at 192 hpe (E, F) first roots with vascular bundles (v) surrounded by elongated cells (ec) of the elongation zone and root hairs (rh). Indicated bars represent 500 µm for A and E, 200 µm for F and 100 µm for B, C and D. (TIF) [file pone.0100997.s001.tif]
