# Supplementary material for: Comprehensive Transcriptome Analysis Unravels the Existence of Crucial Genes Regulating Primary Metabolism during Adventitious Root Formation in Petunia hybrida
Source: PLoS One. 2014 Jun 30;9(6):e100997. doi: 10.1371/journal.pone.0100997 (PMC4076263; doi:10.1371/journal.pone.0100997)
Supplement: Table S1 — Gene-specific primers for Real-Time qPCR. Analysis was carried out using actin as housekeeping gene. All Real-Time qPCR reactions were repeated three times. (DOCX) [file pone.0100997.s002.docx]

**Table S1**.

| **Seq. Id.** | **Function** | **Forward Primer (5’ – 3’)** | **Reverse Primer (5’ – 3’)** |
| --- | --- | --- | --- |
| GO_drpoolB-CL9530Contig1 | Pectinase | CCGTCAGTATTGGGGCTTAG | ACCACACAGGGGACCTAATG |
| cn1111 | Glucose transporter | CCCACTGCTCAGCTGTAAGA | AGAAACGTGAATTCGCAAGG |
| cn8317 | Nitrate transporter | ACAACAAGGCCATGGTTAGC | TCCAATTCAATCCCCATTTC |
| IP_PHBS008L07u | F-box | ACAGCATCAGGGAGACATTG | CAAAACTGTCAGGGTTGGAC |
| IP_PHBS007P04u | Ubiquitin-protein ligase | TGGACAGTGCCAATCAAGTC | CCTCGCAAAGGAAAGTGAAG |
| cn3641 | Trehalose-p-phosphatase | CGAGTGCGTGCTACTCTCTG | CTGCTCCCTAGACCATCTGC |
| cn5371 | Zinc/Iron transporter | GGGATTGCTTAATGCATGTTC | CATGCCACCAGCACCTAGTA |
| cn1159 | Actin | TCAGATTTGCTGGCATGAAG | ATTGTCCAAAGCAAGGATGG |
